# Expression of a Siglec-Fc Protein and Its Characterization

**DOI:** 10.3390/biology12040574

**Published:** 2023-04-10

**Authors:** Kaijun Chi, Huilin Xu, Hanjie Li, Ganglong Yang, Xiaoman Zhou, Xiao-Dong Gao

**Affiliations:** 1 The Key Laboratory of Carbohydrate Chemistry and Biotechnology, Ministry of Education, School of Biotechnology, Jiangnan University, Wuxi 214122, China; 2 State Key Laboratory of Biochemical Engineering, Institute of Process Engineering, Chinese Academy of Sciences, Beijing 100190, China

**Keywords:** sialic acid, Siglec-Fc, glycosylation, protein expression systems

## Abstract

**Simple Summary:**

The Siglec-Fc protein, a fusion protein combining Siglec with the Fc part of a human antibody, is a promising sialic acid-Siglec axis-targeted agent for cancer treatment and is widely used for Siglec ligands discovery. The recombinant Siglec-Fc fusion protein has been expressed in different cell systems. However, its characteristics have not been investigated in detail. In this study, HEK293 and CHO cell lines were used to express the Siglec9-Fc protein, and their adaptability for production was compared. We optimized culture conditions and compared the glycosylation, yield, dimerization and sialic acid binding activity of the Siglec9-Fc protein produced in HEK293 and CHO. Using purified recombinant protein, we further analyzed the distribution of Siglec9 ligands on cancer cell lines, as well as bladder cancer tissue, and revealed the potential ligands. Our findings provide support for the selection of Siglec9-Fc protein expression systems and detection of related Siglec9 ligands.

**Abstract:**

The emerging importance of the Siglec-sialic acid axis in human disease, especially cancer, has necessitated the identification of ligands for Siglecs. Recombinant Siglec-Fc fusion proteins have been widely used as ligand detectors, and also as sialic acid-targeted antibody-like proteins for cancer treatment. However, the heterogenetic properties of the Siglec-Fc fusion proteins prepared from various expression systems have not been fully elucidated. In this study, we selected HEK293 and CHO cells for producing Siglec9-Fc and further evaluated the properties of the products. The protein yield in CHO (8.23 mg/L) was slightly higher than that in HEK293 (7.46 mg/L). The Siglec9-Fc possesses five N-glycosylation sites and one of them is located in its Fc domain, which is important for the quality control of protein production and also the immunogenicity of Siglec-Fc. Our glycol-analysis confirmed that the recombinant protein from HEK293 received more fucosylation, while CHO showed more sialylation. Both products revealed a high dimerization ratio and sialic acid binding activity, which was confirmed by the staining of cancer cell lines and bladder cancer tissue. Finally, our Siglec9-Fc product was used to analyze the potential ligands on cancer cell lines.

## 1. Introduction

Sialic acid-binding immunoglobulin-like lectins (Siglecs) are a family of immunomodulatory receptors expressed in hematopoietic lineages [1]. In humans, 14 Siglecs have been identified, each containing an outmost (N-terminal) V-set Ig domain with a sialic acid binding site, and the majority of these receptors possess inhibitory activity. Upon engaging glycans containing sialic acid (sialoglycans), Siglecs signal via immunoreceptor tyrosine-based inhibitory motifs (ITIMs) within their cytoplasmic domain, which dampens the biological function of immune cells [2].

Hypersialylation has been observed in various types of cancer, likely resulting from the elevated metabolic flux of sialic acid as well as the aberrant expression of sialyltransferases and sialidases [3,4]. Cancer cells exploit this sialic acid–Siglec axis to modulate immune cell function in the tumor microenvironment, thereby evading immune surveillance [5]. To block the interaction between sialic acid and Siglecs, several targeted approaches have been developed, such as global sialylation inhibition, antibody-based targeting strategies, sialic acid mimetics and CAR-T cells [6,7,8].

The Siglec-Fc protein, which is a recombinant antibody-like protein fused in the extracellular domain of the Siglec, combines the N-terminal with the IgG1 antibody in the Fc domain. Therefore, in theory, it possesses both the sialic acid binding activity (mediated by the Siglec) and the antibody Fc domain mediated biological functions such as antibody-dependent cellular cytotoxicity (ADCC). The sialic acid binding activity is mediated by the V-set domain of the Siglec, and the conserved arginine amino acid is critical for such activity [9,10]. The Fc domain contributes to protein stability, prolongs the antibody half-time *in vivo*, and interacts with Fc receptors expressed in immune cells such as the NK cell and the macrophage to assist the clearance of “foreigners” and maintain homeostasis [11]. Therefore, recombinant Siglec-Fc proteins are promising antibody-like proteins for blocking Siglec signaling and aiding tumor clearance [12]. Additionally, they are also used as a means of studying Siglec ligands and facilitating the development of sialic acid-Siglec axis-targeted antibodies and glycan mimetics [13]. For recombinant proteins, especially recombinant antibody production, glycosylation is a critical factor to consider [14,15]. It has been demonstrated that the core-fucosylation, which is fulfilled through catalysis of the unique α-1,6-fucosyltransferase (FUT8) on the N-glycan of the antibody Fc domain, could affect the ADCC activity [16]. Due to the complex protein structure and underlying glycosylation modification, it is difficult for the Siglec9-Fc protein to be expressed in relatively low systems such as *E. coli*. So at present, many mammalian cell protein expression systems have been used to produce Siglec-Fc proteins (e.g., CHO [17], Expi293F [18]). An extensive overview and understanding of the properties of Siglec-Fc proteins produced in distinct systems is still lacking.

In this study, we expressed Siglec9-Fc, a representative Siglec-Fc protein, in HEK293 and CHO cell lines, and optimized culture conditions to increase protein yield. We compared glycosylation, sialic acid binding activity and dimerization of the Siglec9-Fc, and further detected ligands distribution on cancer cell lines of different tissue origin and bladder cancer tissue using produced protein. Our findings demonstrated the differences in glycosylation modifications and other protein properties of Siglec9-Fc produced in distinct systems. These results provide experimental support for choosing the appropriate expression system for Siglec-Fc proteins and detecting potential ligands.

## 2. Materials and Methods

### 2.1. Plasmid Construction and Cell Culturing

The extracellular domain of Siglec9 (1–348) was cloned from human peripheral blood cell (PBMC) cDNA which was kindly provided by Dr. Zhou and the IgG1-Fc domain (99–330) was cloned from pME18Sf-IgLD plasmid, which was kindly provided by Prof. Morihisa Fujita. These two sequences were assembled using Multi-quick cloning kits (Talen-bio; Shanghai, China) and a 6×His tag was linked at the C terminal of the Fc domain for protein purification. Subsequently, the Siglec9_(1–348)_-Fc-6His was ligated into a lentiviral vector pLVX-AcGFP-N1 (Takara; Shanghai, China) using ligation mix (Takara; Shanghai, China). Plasmid was amplified using *E.coli (DH5α)* cultured in an LB medium containing ampicillin. The sequence was confirmed by PCR and DNA Sanger sequencing.

HEK293 (human embryonic kidney 293 cell), HEK293T, A549 (lung cancer), K562 (myeloid leukemia), HepG2 (liver cancer), Hela (cervical cancer), and MCF7 (breast cancer) were cultured in the DMEM medium (Biological Industries; Beit Haemek, Israel) with 10% (*v*/*v*) FBS (Biological Industries; Beit Haemek, Israel) and 1% (*v*/*v*) penicillin/streptomycin (Beyotime; Nantong, China) at 37 °C in 5% CO_2_ atmosphere. T24 (bladder cancer), Jurkat T (lymphocyte leukemia) and CHO (Chinese hamster ovary cell) were cultured in the RPMI 1640 medium (Biological Industries; Beit Haemek, Israel) with 10% (*v*/*v*) FBS and 1% (*v*/*v*) penicillin/streptomycin at 37 °C in 5% CO_2_ atmosphere. All the cell lines were obtained from the Cell Bank of the Type Culture Collection of Chinese Academy of Sciences. The lentivirus infection cell lines were storied in our laboratory.

### 2.2. Construction of Protein-Expressing Cell and Optimization of Culture Conditions 

10^6^ cells/well HEK293T were seeded in a 6-well plate. A total of 4 µg plasmids (2 µg pLVX-Siglec9-Fc, 1 µg pMD2.G and 1 µg psPAX2 (Addgene; Cambridge, MA, USA)) and 10 µL lipofectamine 8000 (Beyotime) were mixed with 0.25 mL Opti-MEM culture medium (Thermo Fisher Scientific; Shanghai, China), respectively. The liquid was blended with plasmids or Lipofectamine 8000 and allowed to rest for 20 min before being added to the HEK293T cells. The cell culture medium was changed after 12 h and the cells were cultured for an additional 48 h. Wild-type HEK293 or CHO cells were incubated with the collected lentivirus medium for 24 h. Cells were placed at 32 °C in a 5% CO_2_ incubator during the lentiviral package and infection process. Stable transfected cells were selected using puromycin (InvitroGen; Shanghai, China) at the concentration of 1 µg/mL for HEK293 cells and 10 µg/mL for CHO cells.

For the optimization of culture conditions, we set different culture condition combinations (FBS concentration (1%, 5%, 10%), culture temperature (32 °C, 37 °C) and culture time (72 h, 96 h, 120 h)), and the product was determined by dot blot. Five independent experiments were conducted on each group of samples.

### 2.3. Protein Production and Purification

3 × 10^6^ stable transfected HEK293 or CHO cells were seeded in a 15 cm cell culture dish. After reaching confluence, cell supernatant was harvested 5 days later and filtered through a 0.22 µm filter. The purified protein was obtained by Ni affinity chromatography (Cytiva; Shanghai, China) according to the manufacturer’s instructions. The purified protein was then dissolved in PBS containing 1% glycerol and stored at −20 °C.

### 2.4. Flow Cytometry (FACS)

10^6^ cells were harvested using collected buffer (2 mM EDTA, 1% BSA, phosphate buffered saline (PBS)) and separated into two groups. One group was incubated with 15 units/mL of sialidase (Yuan ye; Shanghai, China) for 30 min at 37 °C. After sialidase treatment, stained cells with purified Siglec9-Fc (7 μg/mL) and PE-conjugated goat anti-human IgG Fc (InvitroGen; Shanghai, China) for 30 min. The cells were then washed with FACS buffer (PBS containing 1% BSA and 0.1% NaN_3_) and the sample was analyzed using Accuri C6 instrument (BD Biosciences, Lake Franklin, NJ, USA). Data were further processed using FlowJo (v10.8.1) software.

### 2.5. Protein Dimerization and Glycosylation Analysis

For protein dimerization analysis, 2 µg of purified protein was mixed with SDS loading buffer with or without β-mercaptoethanol (ME). The sample with ME was boiled for 5 min while the sample without ME was denatured at 37 °C for 1 h. Then, samples were sent to western blot analysis. For protein glycosylation, 2 µg of purified protein was first reduced with 10 mM of dithiothreitol (DTT) for 45 min at 56 °C, then alkylated with 20 mM iodoacetamide (IAM) for 30 min in the dark at room temperature. The protein was digested with or without 0.5 µg PNGase F (Shengxia; Beijing, China) for 12 h at 37 °C. The SDS loading buffer was added to samples and the western blot was used to analyze the changes in the molecular weight of the protein.

### 2.6. Western Blot and Dot Blot

Protein samples were separated by SDS-PAGE and transferred onto polyvinylidene fluoride (PVDF) membrane (Bio-Rad; Hercules, CA, USA). The dot blot was performed as previously described [19]. In brief, 2 µL cell culture medium from each cell culture condition was dropped onto a nitrocellulose (NC) membrane (Beyotime). A standard curve was made by serial dilution of purified Siglec9-Fc whose concentration was confirmed using a BCA protein assay kit (Beyotime). The membrane was allowed to dry for 1 h at room temperature. Non-specific binding was blocked with 5% milk in TBST, and the PVDF or NC membrane was incubated with an anti-His antibody and corresponding HRP-conjugated secondary antibody (TransGen Biotech, Beijing, China) for 1 h. The grayscale of each dot was measured using Tanon Image software (1.0.0.0).

### 2.7. Immunofluorescence

The method for tissue immunofluorescence was described previously [20]. In brief, slides were baked and dewaxed in 100% xylene, followed by a gradient alcohol wash and antigen retrieval. The slides were then blocked with 5% BSA in PBS and incubated overnight with 200 μL of immunofluorescence buffer containing 7 μg/mL of the HEK293-produced and purified Siglec9-Fc protein. After being washed with PBS wash 3 times, the sections were stained with a PE-conjugated goat anti-human IgG Fc secondary antibody and 4’ ,6-diamidino-2-phenylindole (DAPI; D21490, Invitrogen).

For cell immunofluorescence, cells were seeded onto glass coverslips, followed by a PBS wash and fixation with 4% para-formaldehyde for 10 min at room temperature. The subsequent blocking and staining steps were the same as those for tissue immunofluorescence. For cell immunofluorescence, cell actin was stained using Actin-Tracker Green (Beyotime).

Images were captured with a Nikon C2si confocal microscope using a CFI Plan Apochromat VC oil objective lens and adjusted with ImageJ (2.1.0/1.53c, NIH, Bethesda, MD, USA) software.

### 2.8. Immunoprecipitation MS/MS (IP-MS/MS) and LC-MS/MS Samples Preparation

T24 cells cultured in a 10 cm dish were harvested and lysed with 1 mL RIPA buffer (Beyotime) containing 1% protease inhibitor (Sigma-Aldrich; Shanghai, China) on ice for 30 min. The supernatant was obtained by centrifugation and divided into treatment and control groups. 50 µL of prewashed protein A&G beads (Beyotime) and 20 µg HEK293-produced and purified Siglec9-Fc were added to the treatment group, and an equal amount of protein A & G beads were added to the control group. After mixing at 4 °C for 2 h, the beads were washed with PBS and eluted with 0.2 M glycine-HCl buffer (pH = 3.0) to get proteins; the pH of the solution was then adjusted to 7.0 using 1 M Tris-HCl buffer (pH = 8.5). IP proteins or 20 µg purified Siglec9-Fc were reduced using 10 mM DTT at 56 °C for 45 min and alkylated with 20 mM IAM in the dark for 30 min at room temperature. Sequencing grade trypsin (Shengxia; Beijing, China) was added to the solution at 1:40 (*w*/*w*), followed by incubation overnight at 37 °C. The peptides were desalted using C18 resin pipet tips (ZipTip, Millipore Corp, Billerica, MA, USA).

### 2.9. LC-MS/MS Analysis

Lyophilized peptides were suspended in 2% ACN/0.1% formic acid (FA) solution. The samples were first separated on an EASY-nLC 1200 system (Thermo Scientific, San Jose, CA, USA) with an RSLC C18 column (1.9 µm × 100 µm × 20 cm) packed in house and then coupled with a high-resolution Orbitrap Fusion Lumos spectrometer (Thermo Scientific, San Jose, CA, USA). The mobile phase consisted of water (A), 0.1% FA and 90% ACN (B), and the gradient elution was 2–4% B, 8 min; 4–20% B, 80 min, 20–32% B, 22 min; 32–80% B, 5 min; and 80% B, 5 min. The flow rate was maintained at 500 nL/min. Spectra (AGC target 4 × 10^5^ and maximum injection time 250 ms) were collected from 350 to 2000 *m*/*z* at a resolution of 60,000, followed by data-dependent HCD MS/MS (at a resolution of 30,000, HCD collision energy 32%, stepped collision energy 5%, AGC target 5 × 10^4^, and a maximum injection time 35 ms and microscans 1).

### 2.10. Data Analysis

The acquired MS/MS spectra for Sigelc9-Fc glycan composition analysis were processed using pGlyco3.0 software with a built-in glycan structure database [21]. The acquired MS/MS spectra of IP proteins were searched using MaxQuant software (2.0.1.0) with the Homo sapiens Uniprot-organism FASTA database (May 2022) [22]. The results were visualized by R (4.0.3) and GraphPad Prism (8.3.0). The heatmap figures were prepared using the R ComplexHeatmap package (2.6.2) and GO enrichment was prepared using the clusterProfiler package (3.18.1). Comparisons between two groups were performed using the unpaired two-tailed Student’s *t*-test, values of *p* < 0.05 were considered statistically significant.

## 3. Results

### 3.1. Optimizing Culture Conditions to Increase Siglec9-Fc Protein Yield in Mammalian Cells

**Figure 1:**
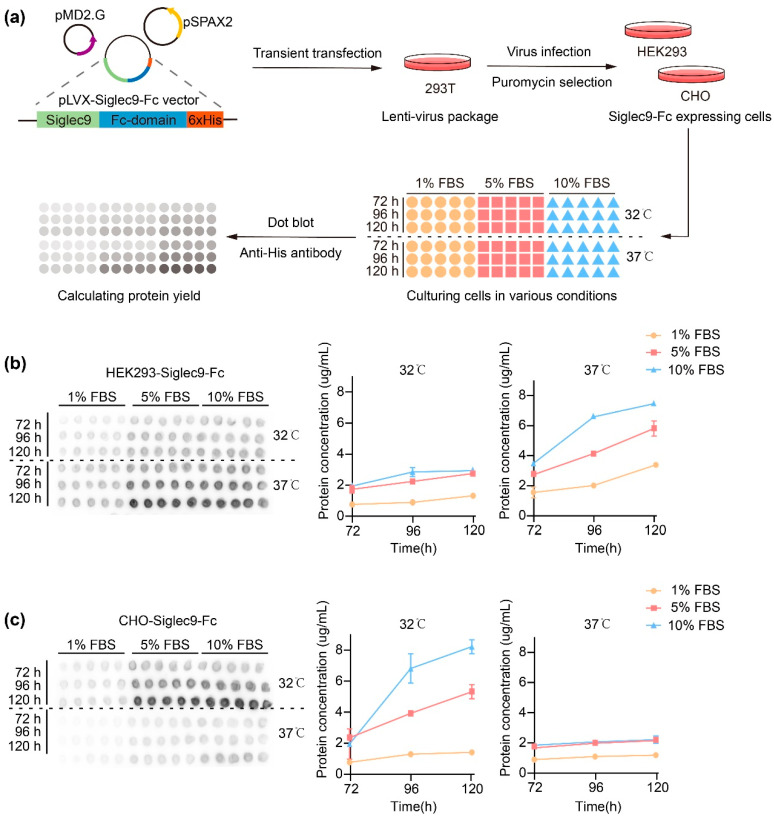
Optimizing culture conditions in mammalian cells. (**a**) Workflow of Siglec9-Fc expressing cell construction and culture condition optimization; (**b**) The results of dot blot and protein yield quantification in HEK293 cells, detected by anti-His antibody; (**c**) The results of dot blot and protein yield quantification in CHO cells, detected by anti-His antibody.

The Siglec9-Fc-expressing HEK293 or CHO cells were obtained using lentiviral infection and puromycin selection (Figure 1a). The recombinant protein fused the human Siglec9 extracellular domain with the Fc part of human IgG1. A 6×His tag was added at the C-terminal of the recombinant protein for detection and purification. A second-generation lentiviral system (three plasmids) and HEK293T cell were applied to the lentivirus package. In order to obtain higher protein yield and determine the best cell culture conditions, we took the culture temperature (32 °C, 37 °C), FBS concentration (1%, 5%, 10%) and culture time (3 d, 4 d, 5 d) into consideration. Initially, an equal number of cultured cells were kept in different culture conditions and collected the cell culture medium for further protein yield analysis. The targeted protein level in each situation was acquired by dot blot and anti-His antibody. The protein quantification was achieved using a standard curve (Figure S1a,b). As shown in Figure 1b, for protein expression in HEK293, the average protein yield at 37 °C was higher than that at 32 °C at each FBS concentration, and the yield rose with the increase of FBS concentration at each culture temperature. On the other hand, the average production in CHO was almost the same at 1% FBS concentration both at 32 °C and 37 °C. In CHO, the average protein yield at 10% FBS was higher than that at 5% FBS cultured in 32 °C, while it remained the same at 37 °C (Figure 1c). Finally, the highest yield of Siglec9-Fc in HEK293 rose up to 7.46 mg/L, and the optimal culture condition combination was 10% FBS, 37 °C for 5 d. And for CHO, the highest protein yield was 8.23 mg/L, which was a little higher than HEK293, and the optimal culture combination was 10% FBS, 32 °C for 5 d.

### 3.2. Protein Purification and Characterization

**Figure 2:**
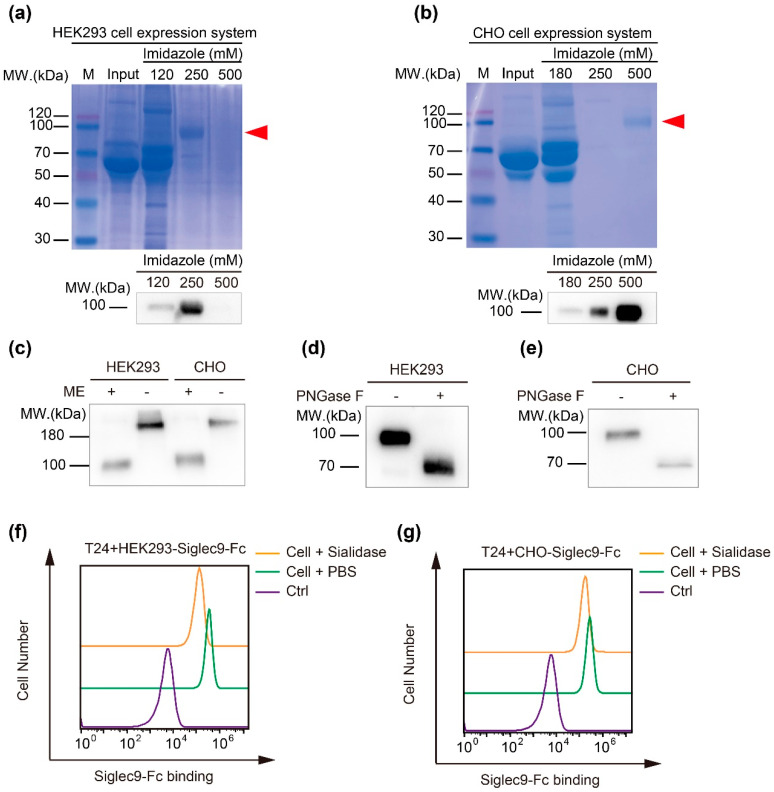
Siglec9-Fc protein purification, dimerization and sialic acid binding activity. Coomassie-stained SDS-PAGE gel and western blot of purified Siglec9-Fc produced in HEK293 (**a**) and CHO (**b**) cell lines, detected by anti-His antibody; (**c**) the western blot of monomer or dimer molecular weight of Siglec9-Fc produced in HEK293 and CHO cell lines, detected by anti-His antibody, ME: β-Mercaptoethanol; the western blot of Siglec9-Fc molecular weight change with PNGase F treatment in HEK293 (**d**) or CHO (**e**), detected by anti-His antibody, PNGase F: Peptide-N-Glycosidase F; the intensity of Siglec9-Fc produced in HEK293 (**f**) and CHO (**g**) binding on bladder cancer cell T24 membrane with or without sialidase treatment.

We cultured Siglec9-Fc expressing HEK293 and CHO in 10% FBS at 37 °C and 10% FBS at 32 °C, respectively. The cell culture medium was harvested 5 days later, and the targeted protein was purified by Ni affinity chromatography. The purity of the targeted protein was over 90% after purification and the molecular weight was about 100 kDa in both HEK293 (Figure 2a) and CHO (Figure 2b) without protein degradation. We then used western blot and flow cytometry to analyze protein dimerization, glycosylation and sialic acid binding activity.

Disulfide bonds are essential for antibody Fc domain to form dimers, which can enhance the stability of antibodies in the physiological environment and also enhance the ADCC effect [11,23]. To investigate the formation of dimerization, we treated Siglec9-Fc protein with or without β-Mercaptoethanol (ME), which can destroy the disulfide bond within the protein. The molecular weight of monomer or dimer was analyzed using western blot. The result showed that the Siglec9-Fc we produced was absolutely dimeric and the molecular weight was about 200 kDa in both HEK293 and CHO (Figure 2c). For glycosylation detection, we treated equal amounts of the denatured Siglec9-Fc protein with PNGase F, which could cut off all N-glycans from the protein. Results could be visualized through molecular weight change using western blot. The monomer molecular weight of Siglec9-Fc changed from 100 kDa to 70 kDa after PNGase F treatment in both HEK293 (Figure 2d) and CHO (Figure 2e). For practical reasons, we used cell-based Siglec9-Fc binding analysis to verify the sialic acid binding activity of targeted protein. We treated the bladder cancer cell line T24 with sialidase, which could cut α-2, 3 or 2, 6-linked sialic acid from glycan and compared the amount of binding Siglec9-Fc on the cell surface using flow cytometry analysis. Compared with the PBS-treated group, the signal intensity of the sialidase-treated group decreased in both HEK293 (Figure 2f) and CHO (Figure 2g).

### 3.3. Analysis of N-Glycosylation on the Recombinant Siglec9-Fc Protein

Glycosylation is an essential quality control factor during manufacturing that can influence the biological function and immunogenicity of antibodies [24]. From the PNGase F treatment results, glycosylation accounted for 30% of Siglec9-Fc molecular weight in both HEK293 (Figure 2d) and CHO (Figure 2e).

**Figure 3:**
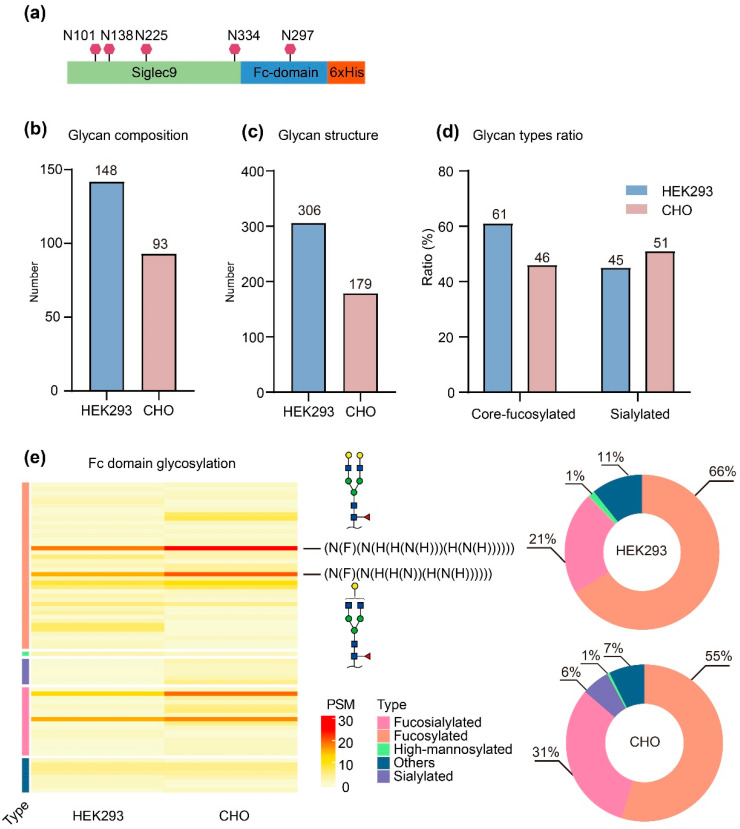
Glycosylation modification of Siglec9-Fc produced in HEK293 and CHO. (**a**) The graph shows five detected glycosylation sites of Siglec9-Fc; the mass results of total glycan compositions (**b**) and potential glycan structures (**c**) detected in HEK293 and CHO; (**d**) the proportion of core-fucosylated or sialylated glycan modification of Siglec9-Fc produced in HEK293 and CHO; (**e**) glycan compositions, distribution and glycan proportion in the Fc domain of Siglec9-Fc protein produced in HEK293 and CHO.

To reveal their glycosylation features, we utilized mass spectrometry to analyze the glycosylation modifications of Siglec9-Fc produced in HEK293 and CHO. The targeted protein was initially reduced and alkylated, followed by enzyme digestion. The desalted glycopeptides were then analyzed with a high-resolution Orbitrap Fusion Lumos spectrometer. After spectrum identification, we confirmed the presence of five glycosylation sites in both HEK293 and CHO, four sites in the Siglec9 part and one in the Fc domain (Figure 3a). The N101 site located in the V-set domain was predominantly glycosylated, and glycan H(5)N(4)A(1) and H(5)N(4)A(1)F(1) were the primary glycan compositions in both HEK293 and CHO (Figures S2 and S3). The N-glycosylation heterogeneity of the Siglec9-Fc produced in HEK293 was found to be higher than that in CHO, as evidenced by the glycan composition (Figure 3b) and the corresponding glycan structure (Figure 3c). Specifically, the amount of identified N-glycan composition of Siglec9-Fc glycopeptides produced in HEK293 was 148, while it was 93 in CHO (Figure 3b). The corresponding amount in the glycan structure was 306 in HEK293 and 179 in CHO (Figure 3c). Among all the glycan modifications on the antibodies, it had been reported that a greater amount of core-fucosylation is associated with a decrease in ADCC effect, while higher sialylation prolongs the half-life period [16]. Therefore, we analyzed these two kinds of glycan modification in Siglec9-Fc produced in HEK293 and CHO, respectively. The results revealed that Siglec9-Fc was highly core-fucosylated in HEK293, whereas it was more sialylated in CHO (Figure 3d). Furthermore, we analyzed the N-glycosylation status of the Fc domain (N297 in Figure 3a). The glycan H(5)N(4)F(1) and H(5)N(5)F(1) were the primary glycan compositions in the Fc domain in both HEK293 and CHO, with higher sialylation in CHO and more fucosylation in HEK293 (Figure 3e). These results indicated that the CHO cell may be the better system for producing recombinant antibody-like proteins with higher ADCC activity and a longer half-life period.

### 3.4. Immuno-Blotting Siglec9 Ligands on the Cell Membrane of Cancer Cells and Tissue Using Siglec9-Fc

**Figure 4:**
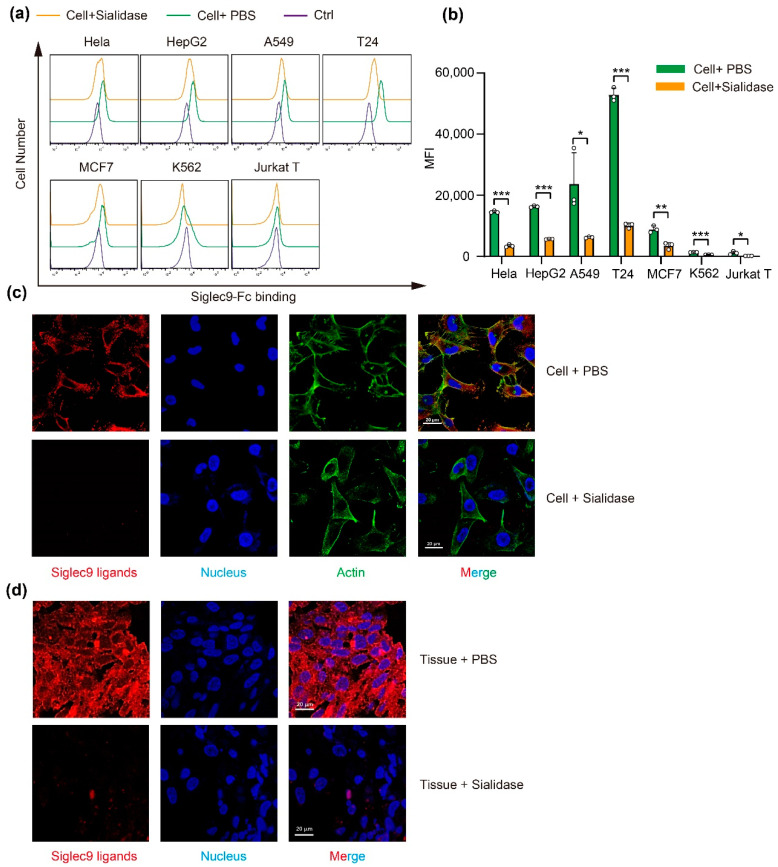
Siglec9 ligands widely expressed on cancer cell lines and bladder cancer tissue. (**a**) Flow cytometry analysis of Siglec9-Fc binding on cancer cells of different tissue origin before or after sialidase treatment; (**b**) quantification of flow cytometry analysis of the Siglec9-Fc binding. Data presented as mean values ± SD. Statistics: unpaired two-tailed Student’s t-test (* *p* < 0.05, ** *p* < 0.01, and *** *p* < 0.001); Siglec9-Fc binding intensity on the bladder cancer cell T24 (**c**) and bladder cancer tissue (**d**), which decreased after sialidase treatment shown by confocal microscopy. Red: Siglec9 ligands, stained by Siglec9-Fc and PE-anti human IgG, Blue: nucleus, stained by DAPI, Green: actin, stained by Actin-Tracker Green.

The Siglec-Fc recombinant protein is an ideal agent in which to invest the ligands of related Siglecs. In our experiment, we used Siglec9-Fc produced in the HEK293 to test Siglec9 ligands on various cancer cells surface from different tissues. Cancer cells pretreated with sialidase or PBS were stained with the Siglec9-Fc protein and a fluorescently labeled secondary antibody, which was then analyzed for differences in fluorescence intensity. The signal intensity suggested that Siglec9-Fc ligands were present on all cancer cells. In addition, the binding amount decreased after sialidase treatment compared with the PBS treated group, as determined by flow cytometry (Figure 4a,b). Among the tested cells, the bladder cancer cell T24 had the highest binding intensity, whereas the binding signal was relatively low on myeloid leukemia K562 and lymphocyte leukemia Jurkat T cells (Figure 4a,b). To figure out the ligands distribution characteristic, we visualized the Siglec9 ligands distribution on bladder cancer cells T24 as well as bladder cancer tissue. The confocal imaging results showed that the Siglec9-Fc signal mainly presented on the cell membrane (shown in red) and the binding signal almost disappeared after sialidase treatment in both T24 cells (Figure 4c) and bladder cancer tissue (Figure 4d).

### 3.5. Siglec9 Ligands Detection

**Figure 5:**
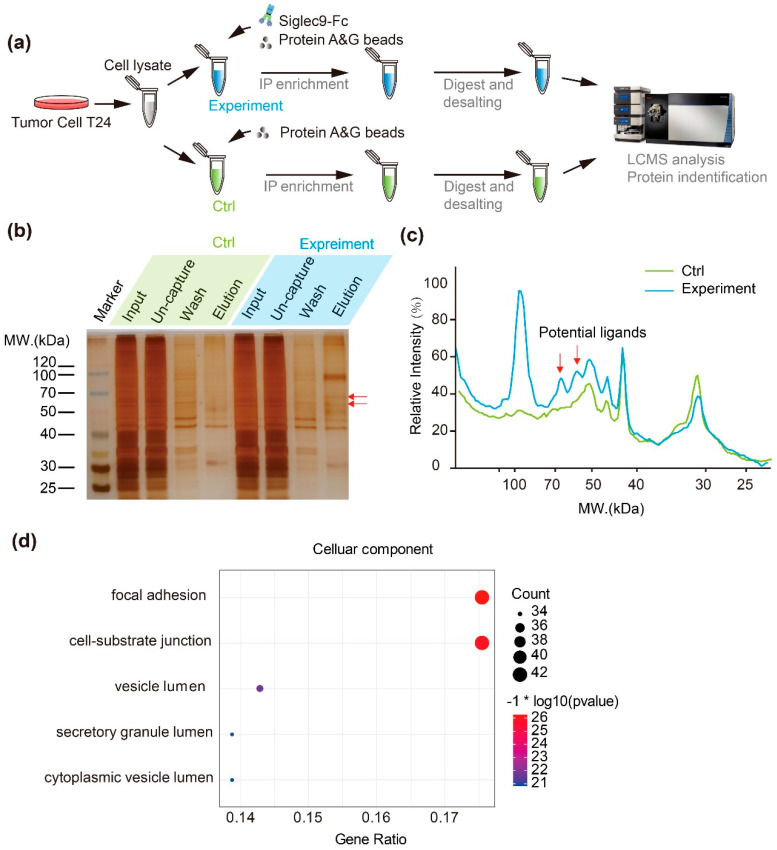
Siglec9 ligands on T24 bladder cancer cell. (**a**) Graphical representation of the steps for the Siglec9-Fc IP-MS/MS experiment process; (**b**) silver staining result of Siglec9-Fc potential ligands; (**c**) corresponding intensity of Siglec9-Fc potential ligands’ bands which localized between 50 kDa-70 kDa, (blue or green line represented the silver staining bands intensity in ctrl or experiment group and the potential ligands localization was marked with red arrows.); (**d**) enriched cellular component bubble plot of potential Siglec9-Fc interaction proteins.

The T24 bladder cancer cell line, which exhibited the highest Siglec9-Fc binding intensity (Figure 4a,b), was selected for the IP-MS/MS experiment to identify potential Siglec9 ligands. The T24 cell lysate was divided into treatment and control groups. Equal amounts of protein A&G beads which could capture the Fc domain of antibodies were added to each group. The purified HEK293-produced Siglec9-Fc protein was added to the experiment group to pull down the potential ligands, which were identified by LC-MS/MS analysis (Figure 5a). First, we used silver staining SDS-PAGE (Figure 5b) and coomassie staining SDS-PAGE (Figure S5) to visualize the Siglec9 ligands’ molecular weight distribution. Compared with the control group, the unique bands of potential Siglec9 ligands were localized between 50–70 kDa, which were indicated by the red arrows (Figure 5c). After data confidence filtering and subtracting the same proteins detected in the control group, we identified 301 proteins in the experiment group (Table S3). Then we did protein location and functional analysis, and results indicated that the enriched cellular components were concentrated on focal adhesion and cell-substrate junction (Figure 5d). The proteins involved in these two cellular components and related signaling pathways need to be investigated in order to figure out the biological consequence after Siglec9 and ligands’ engagement. Among all the detected results, basigin, integrin beta, CD44 antigen, and transmembrane emp24 domain-containing protein 10 were reported as glycoproteins which localized on the cell membrane. However, the glycosylation situation of these proteins is not fully illustrated and their interactions with Siglec9 *in vitro* or *in vivo* still need to be invested. In order to compare the ligands’ binding with that of Siglec9-Fc produced in HEK293 and CHO, we also used Siglec9-Fc produced in CHO to do the IP experiment. The identified proteins’ correlation coefficient was up to 83% (Figure S4), and all the identified proteins were listed in Table S4. Some glycoproteins such as the galectin-3-binding protein (LGALS3BP) and galectin-1 (LGALS1) were identified in each group. But there remained some unique identified proteins in each group. For example, in the group that used the HEK293-produced Siglec9-Fc protein, we identified glycosylated protein neural cell adhesion molecule 2 (NCAM2), while in the group that used CHO-produced Siglec9-Fc, glycosylated protein fibrinogen alpha chain (FGA) was identified. It has been reported that N-glycosylation modification of CD22 (Siglec2) regulates its binding activity [25,26]. We also revealed that the glycosylation modification of Siglec9-Fc produced in HEK293 and CHO cells was distinct (Figure 3). Therefore, the distinct glycosylation modification of Siglec9-Fc produced in HEK293 or CHO cells may cause the difference in ligands.

## 4. Discussion

Targeting the sialic acid–Siglec axis in tumor microenvironment is an emerging and promising strategy for cancer treatment [6,27]. Among all these targeting materials, Siglec-Fc is a special one owing to its endogenous sialic acid binding specificity and Fc mediated immune activity, such as antibody-dependent cellular cytotoxicity (ADCC) [12]. Additionally, it has been used to elucidate the underlying ligands for Siglecs, which facilitated the development of targeted Siglec ligands antibodies or ligands mimetics [28]. To produce Siglec-Fc, various protein expression systems have been employed. Here, we used HEK293 and CHO to produce Siglec9-Fc and analyzed its properties especially glycosylation modification and potential Siglec9 ligands.

More than 50% of therapeutic proteins on the market are produced in mammalian cells, and glycosylation modification is one of the essential traits of pharmaceutical proteins [24]. In HEK293, higher core-fucosylation was detected on Siglec9-Fc while there was greater sialylation modification in CHO cells. It has been reported that increased sialylation of the Fc domain could prolong antibody half-life, while increased core-fucosylation may decrease ADCC effect [29]. Additionally, the protein yield in CHO cells was higher than HEK293. Considering these two factors, it seems that CHO cell line may be a better system for producing the Siglec9-Fc protein with longer half-time and better ADCC efficacy. However, other aspects should be noted. Unlike human cell lines, CHO can produce N-Acetylneuraminic Acid (Neu5Ac) and hydroxylated derivative N-Glycolylneuraminic acid (Neu5Gc), while in humans there is only Neu5Ac [30], which were also detected in our MS/MS results (Tables S1 and S2). Additionally, injection of therapeutic proteins containing Neu5Gc could induce immune responses to some extent, which may negatively impact the pharmacokinetics or biological activity (efficacy) of therapeutic proteins [31,32]. Meanwhile, in pharmaceutical protein production, one of the sources of Neu5Gc is fetal bovine serum. To eliminate such influence, a serum-free culture method and specific cell lines are widely used in industrial production [33]. Whether serum is used or not, further cellular level and *in vivo* experiments are still needed to compare and monitor efficacy and underlying immunogenicity of Siglec9-Fc produced in different systems.

Siglec interacts with sialic acid-containing glycoconjugates, which can be either on the same (*cis*) cell or opposing (*trans*) cell [34]. This may be the reason why hematopoietic linage Jurkat T and K562 cell lines had relatively low binding signal. When it comes to Siglec9 ligands’ detection, from a traditional point of view, the interaction between Siglec9 and its ligands depends on sialylation modification on the ligands. Some membrane glycoproteins coded by *BSG*, *ITGB1*, *CD44* genes were identified, and they may interact with Siglec9 (Table S3). These proteins are also known to facilitate bladder cancer progression [35,36,37]. Further verification is needed as to whether Siglec9 is also involved in promoting the tumor progression process of such proteins. . Additionally, some studies give a different perspective, and in these cases the interaction is sialic acid-independent. For example Siglec9 bound to the glycosaminoglycan hyaluronic acid (GlcNAcβ1-4GlcAβ1-3)n without sialic acid [38]. And SiglecE, which is a Siglec9 homologous protein, interacted with CD36 in a sialic acid-independent manner [39]. In our IP experiment, we also identified some membrane non-glycosylated proteins, such as Annexin. The function of such non-glycosylated proteins in the interaction between Siglec9 and its ligands still needs to be fully studied. Furthermore, we also detected some secreted glycosylated proteins, such as galectin-1 encoded by LGALS1 and the galectin-3-binding protein encoded by LGALS3BP. At first, they did not attract our attention because their cell localization does not conform to the view of traditional Siglec9 ligands. However, some researchers also showed that LGALS3BP acted as a tumor-associated immunomodulatory ligand for Siglec9 [40]. Therefore, we can also focus on such secreted glycosylated proteins and figure out their roles in Siglec9-mediated biological functions. Considering all of these inquiries and our experiment results, further experimental validation is urgently needed to carefully uncover the interactions between Siglec9 and these identified proteins and figure out their biological consequences in the bladder cancer microenvironment.

## 5. Conclusions

Our results suggest that CHO and HEK293 are suitable for producing Siglec9-Fc, and CHO is better than HEK293 for producing a higher ADCC activity and longer half-life antibodies. Moreover, our study provides experimental evidence for the selection of Siglec-Fc protein expression systems as well as for related Siglec ligands’ detection.

## Data Availability

The data supporting the findings of this study are available within the Article and its Supplementary Information. Further relevant data are available from corresponding authors upon reasonable request.

## Supplementary Materials

The following supporting information can be downloaded at: https://www.mdpi.com/article/10.3390/biology12040574/s1, Figure S1: Siglec9-Fc protein yield calculation standard curves; Figure S2: Glycosylation modification of Siglec9-Fc produced in HEK293 and CHO; Figure S3: MS/MS spectra of Siglec9-Fc glycopeptides producing in HEK293 and CHO; Figure S4. Identified IP experiment proteins’ correlation coefficient using Siglec9-Fc producing in HEK293 or CHO; Figure S5: Siglec9-Fc ligands on T24 bladder cancer cell; Figure S6: Raw data of western blot and dot blot graphs; Figure S7: Flow cytometry analysis of Siglec9-Fc binding on cell surface, raw data of Figure 5a; Figure S8: Flow cytometry analysis of Siglec9-Fc binding on cell surface, raw data of Figure 2d and Figure 5a; Table S1: HEK293-Siglec9-Fc glycopeptides MS/MS result; Table S2: CHO-Siglec9-Fc glycopeptides MS/MS result; Table S3: Siglec9-Fc-IP-MS/MS result; Table S4: HEK293-Siglec9_CHO-Siglec9-IP_MS results.
