# Supplementary material for: Expression of a Siglec-Fc Protein and Its Characterization
Source: Biology (Basel). 2023 Apr 10;12(4):574. doi: 10.3390/biology12040574 (PMC10135921; doi:10.3390/biology12040574)
Supplement: Supplementary file 1 [file biology-12-00574-s001.zip › biology-2293527-Supplementary Figures.pdf]

Expression of a Siglec-Fc protein and its characterization

Kaijun Chi [1], Huilin Xu [1], Hanjie Li [1], Ganglong Yang [2], Xiaoman Zhou [1,*], and Xiao-Dong Gao[1,2,*]

1 The Key Laboratory of Carbohydrate Chemistry and Biotechnology, Ministry of Education, School of Biotechnology, Jiangnan University, Wuxi 214122, China

2 State Key Laboratory of Biochemical Engineering, Institute of Process Engineering, Chinese Academy of Sciences, Beijing 100190, China

* Correspondence: Xiao-Dong Gao: xdgao@ipe.ac.cn; Xiaoman Zhou: xiaoman@jiangnan.edu.cn

Supplementary Data

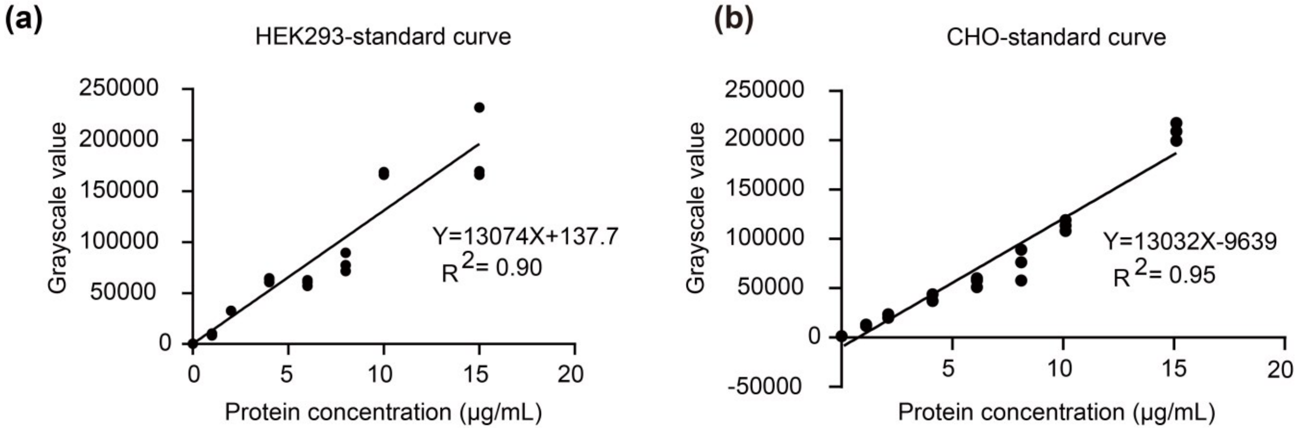

Figure S1: Siglec9-Fc protein yield calculation standard curves. (a) The standard curve for Siglec9-Fc protein yield calculation in HEK293; **(b)** The standard curve for Siglec9-Fc protein yield calculation in CHO.

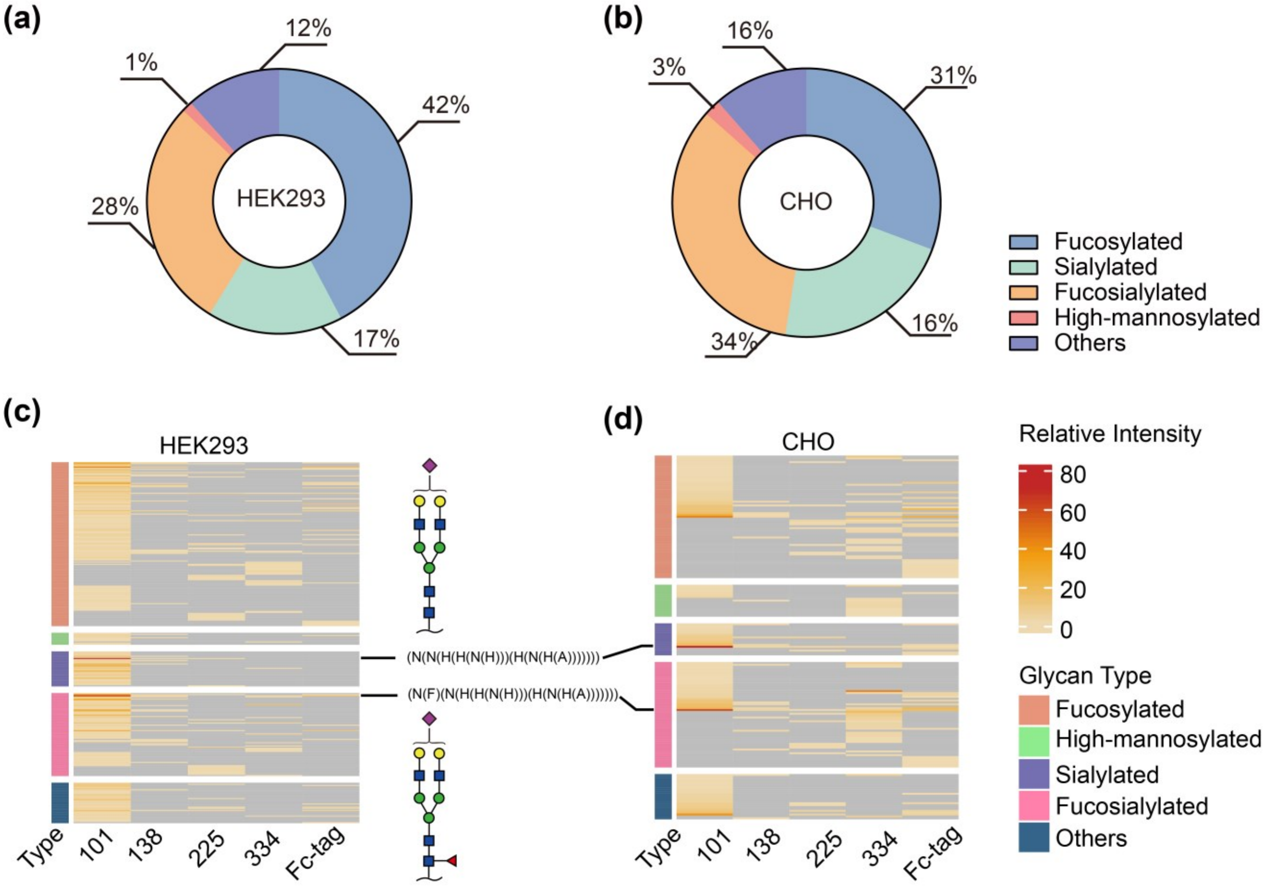

Figure S2: Glycosylation modification of Siglec9-Fc produced in HEK293 and CHO. The summary of the proportion of five types (fucosylated, high-mannosylated, sialylated, fucosialylated and others) of Siglec9-Fc glycosylation produced in HEK293 (**a**) and CHO (**b**); Heatmap of glycan compositions and their amount distribution at five glycosylation sites of Siglec9-Fc produced in HEK293 (**c**) and CHO (**d**).

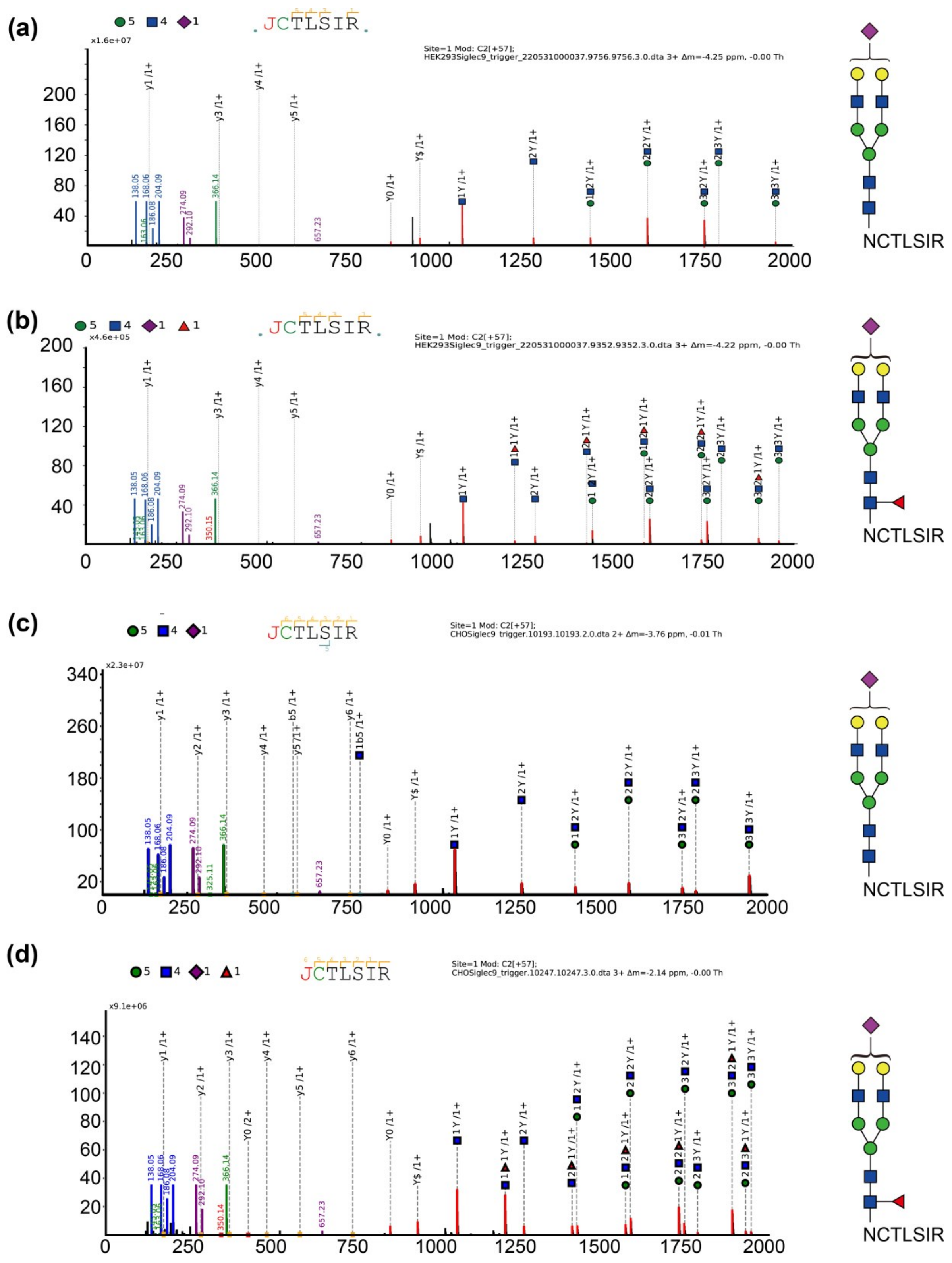

Figure S3: MS/MS spectra of Siglec9-Fc glycopeptides producing in HEK293 and CHO.
The representative MS/MS spectra of Siglec9-Fc glycopeptides N(H(5)N(4)A(1))CTLSIR (**a**) and N(H(5)N(4)A(1)F(1))CTLSIR (**b**) produced in HEK293; The representative MS/MS spectra of Siglec9-Fc glycopeptides N(H(5)N(4)A(1))CTLSIR (**c**) and N(H(5)N(4)A(1)F(1))CTLSIR (**d**) produced in CHO.

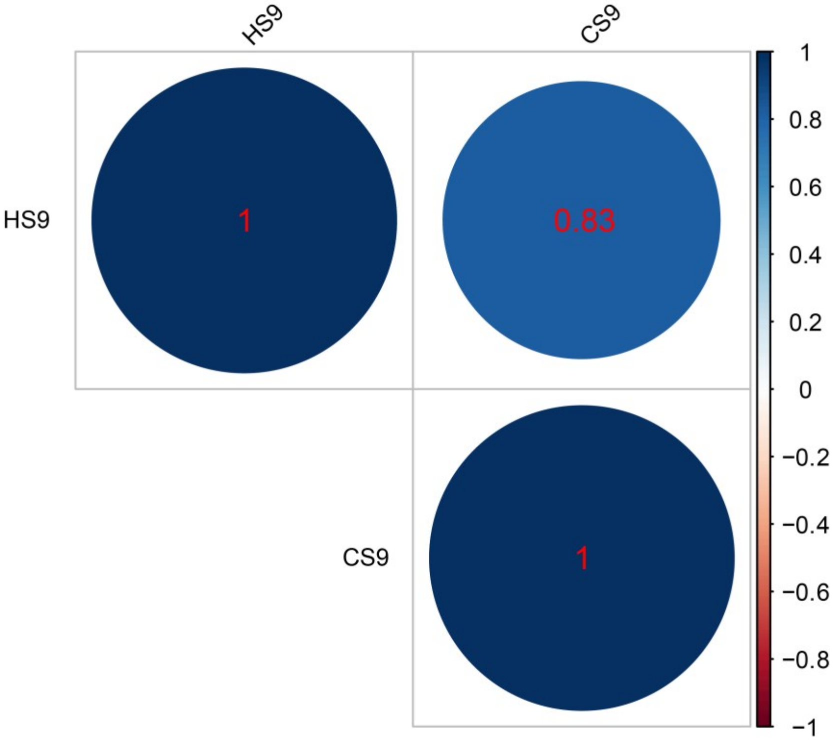

Figure S4. Identified IP experiment proteins' correlation coefficient using Siglec9-Fc producing in HEK293 or CHO. HS9: Siglec9 produced in HEK293, CS9: Siglec9 produced in CHO.

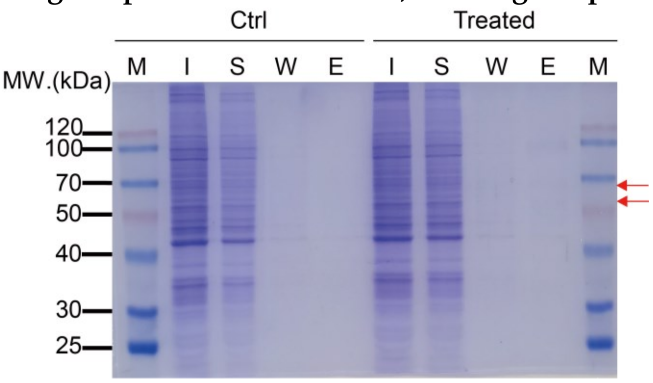

Figure S5. Siglec9-Fc ligands on T24 bladder cancer cell. Coomassie stained SDS-PAGE result of Siglec9-Fc potential ligands, I: input, S: supernatant, W: wash solution, E: elution solution.

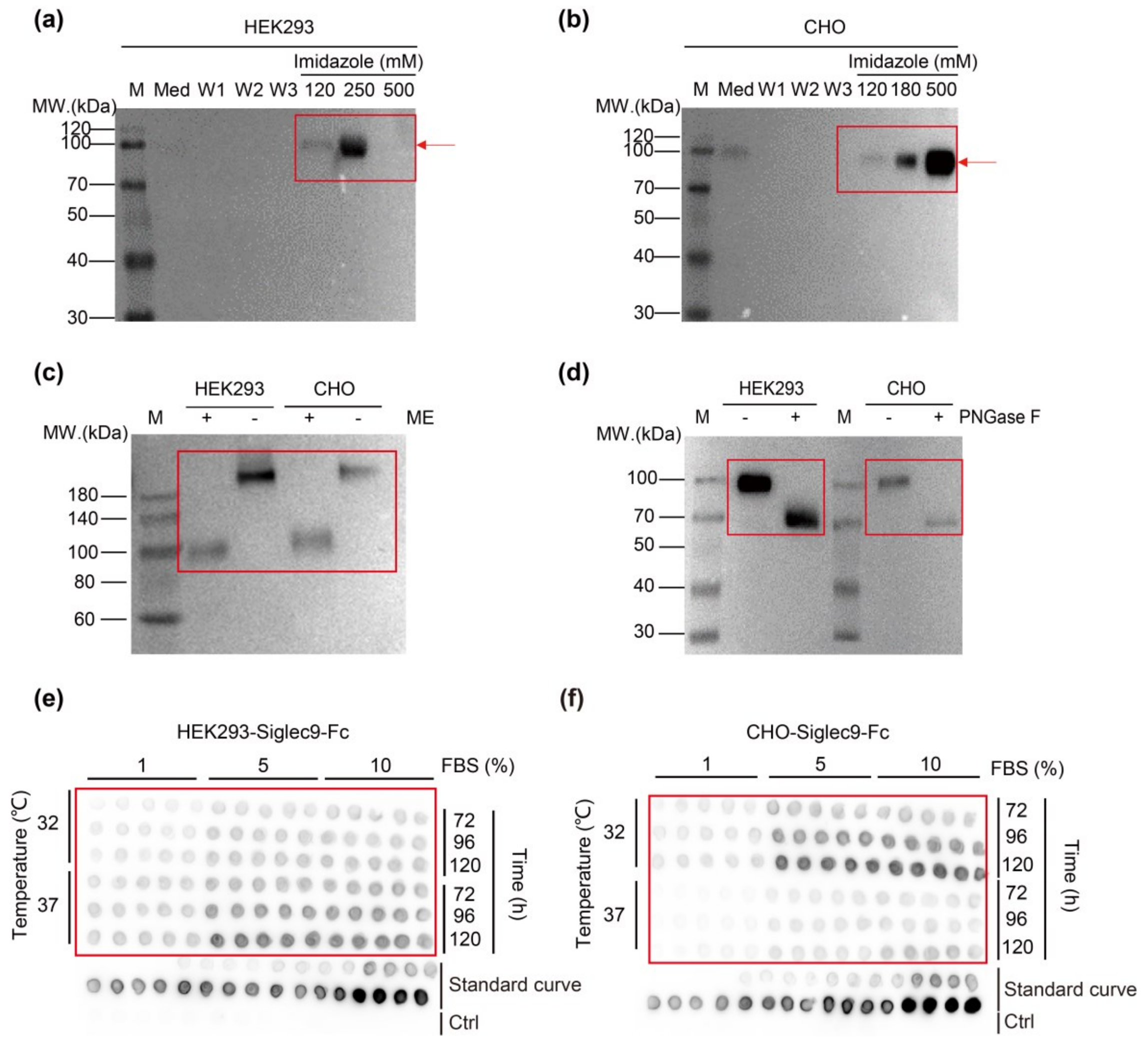

Figure S6: Raw data of western blot and dot blot graphs. (a) Raw data of Figure 2a, Med: Medium, W: Wash; **(b)** Raw data of Figure 2b, Med: Medium, W: Wash; **(c)** Raw data of Figure 2c, ME: β-Mercaptoethanol; **(d)** Raw data of Figure 2d and 2e, PNGase F: Peptide-N-Glycosidase F; **(e)** Raw data of Figure 1b; **(f)** Raw data of Figure 1c.

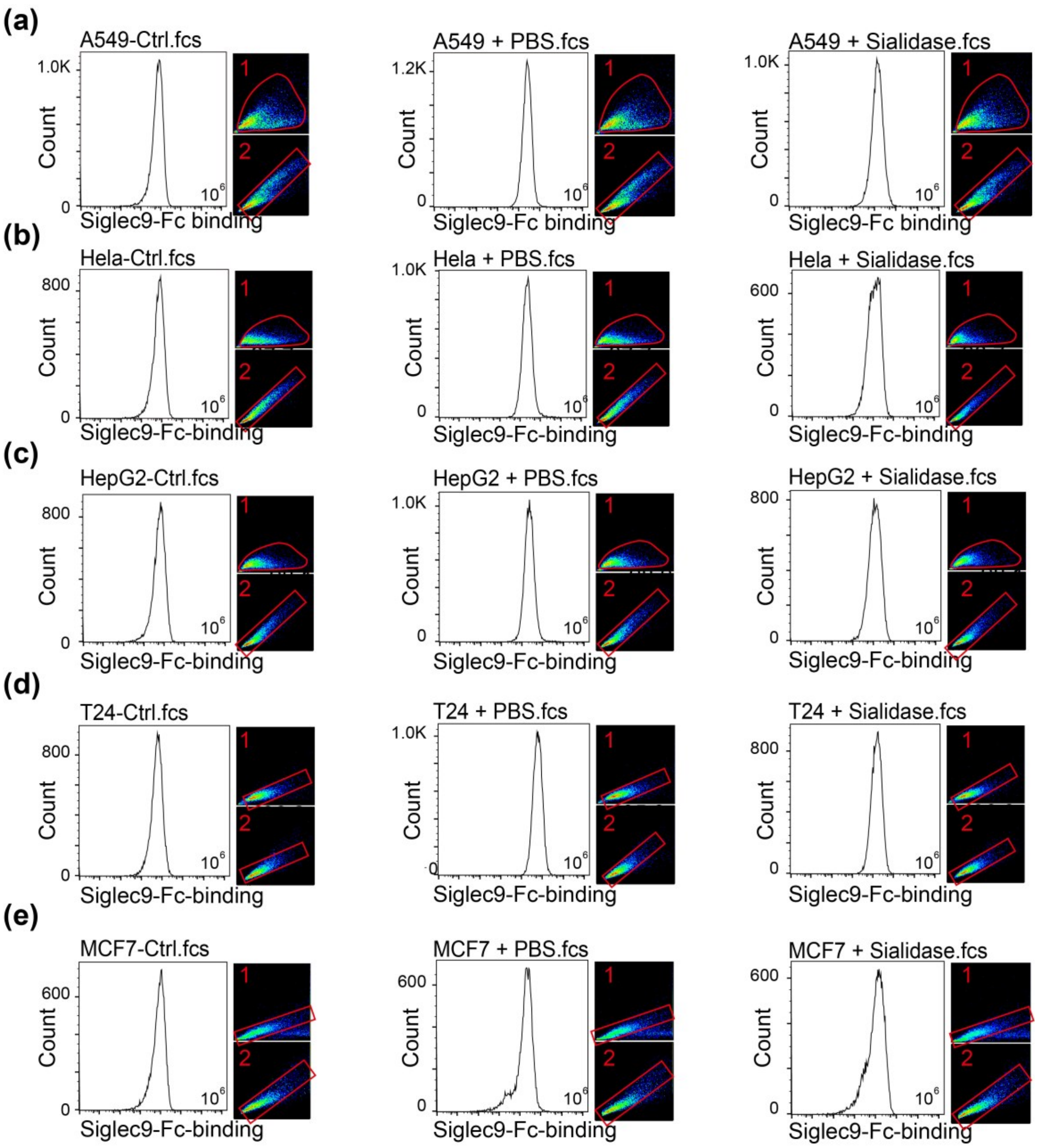

Figure S7. Flow cytometry analysis of Siglec9-Fc binding on cell surface, raw data of Figure 4a. **(a)** Siglec9-Fc binding on A549 cell (lung cancer); **(b)** Siglec9-Fc binding on Hela cell (cervical cancer); **(c)** Siglec9-Fc binding on HepG2 cell (liver cancer); **(d)** Siglec9-Fc binding on T24 cell (bladder cancer); **(e)** Siglec9-Fc binding on MCF7 cell (breast cancer).

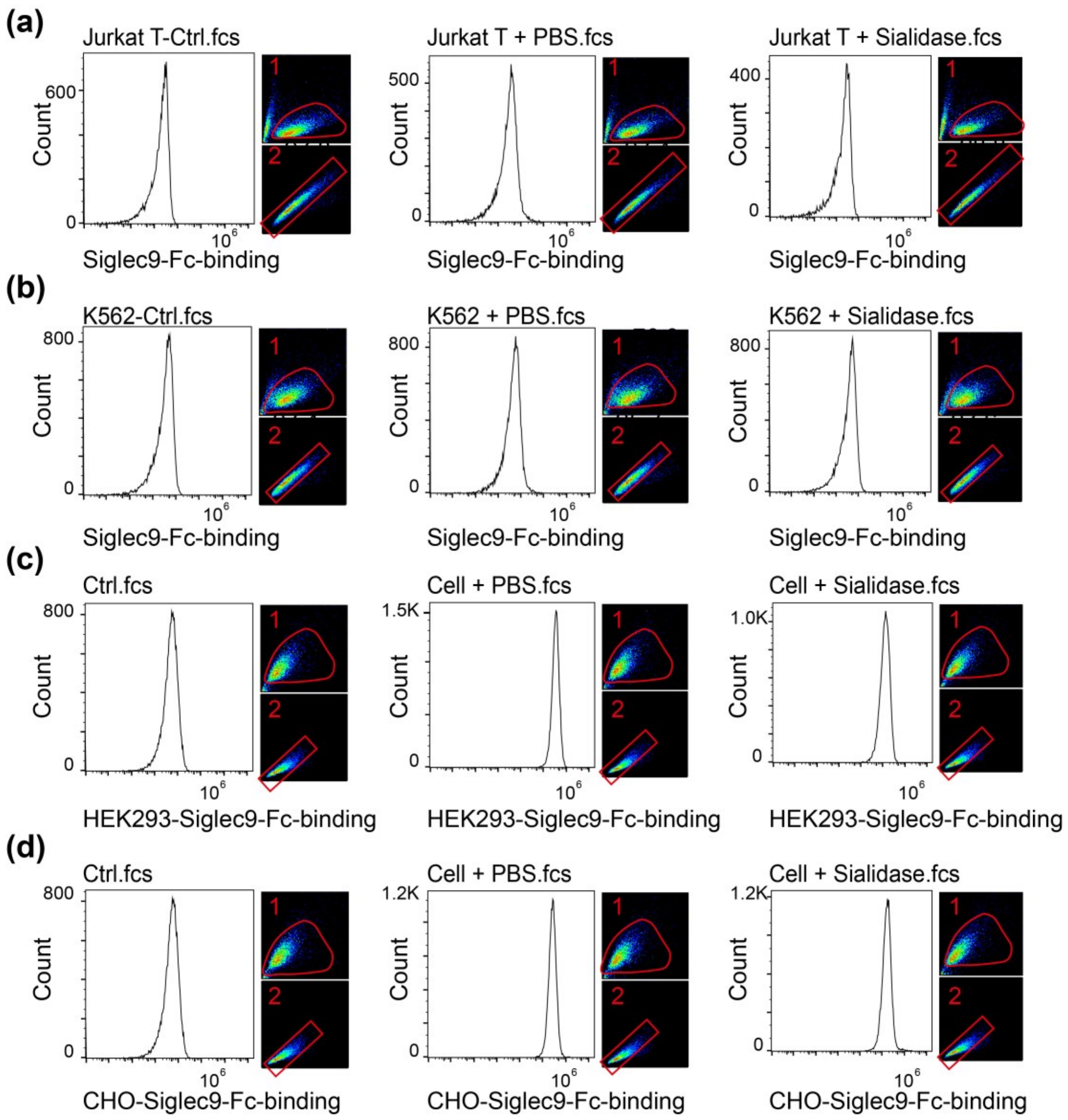

Figure S8. Flow cytometry analysis of Siglec9-Fc binding on cell surface, raw data of Figure 4a, 2f and 2g. (a) Siglec9-Fc binding on Jurkat T cell (lymphocyte leukemia); **(b)** Siglec9-Fc binding on K562 cell (myeloid leukemia); Siglec9-Fc producing in HEK293 **(c)** and CHO **(d)** binding on T24 cell (bladder cancer).
